# Bionic Optimization Design and Fatigue Life Prediction of a Honeycomb-Structured Wheel Hub

**DOI:** 10.3390/biomimetics9100611

**Published:** 2024-10-09

**Authors:** Na Liu, Xujie Liu, Yueming Jiang, Peng Liu, Yuanyuan Gao, Hang Ding, Yujun Zhao

**Affiliations:** 1 Shandong Jianzhu University, 1000 Fengming Road, Lingang Development Zone, Jinan 250000, China; 2 Beijing University of Posts and Telecommunications, No.10 Xitucheng Road, Beijing 100876, China; 3 Jinan University, No.336 South Xinzhuang West Road, Jinan 250000, China; 4 Jinan Lingong Mining & Rock Technology Co., Ltd., Jinan 250000, China

**Keywords:** finite element analysis, response surface optimization design, bionic design, fatigue life prediction

## Abstract

The wheel hub is an important component of the wheel, and a good hub design can significantly improve vehicle handling, stability, and braking performance, ensuring safe driving. This article optimized the hub structure through morphological aspects, where reducing the hub weight contributed to enhanced fuel efficiency and overall vehicle performance. By referencing honeycombed structures, a bionic hub design is numerically simulated using finite element analysis and response surface optimization. The results showed that under the optimization of the response surface analytical model, the maximum stress of the optimized bionic hub was 109.34 MPa, compared to 119.77 MPa for the standard hub, representing an 8.7% reduction in maximum stress. The standard hub weighs 34.02 kg, while the optimized hub weight was reduced to 29.89 kg, a decrease of 12.13%. A fatigue analysis on the optimized hub indicated that at a stress of 109.34 MPa, the minimum load cycles were 4.217 × 10^5^ at the connection point with the half-shaft, meeting the fatigue life requirements for commercial vehicle hubs outlined in the national standard GB/T 5334-2021.

## 1. Introduction

The wheel hub is a component of an automobile that serves the roles of support, steering, driving, and braking [1]. The design of a wheel hub must consider performance, safety, comfort, and compatibility with the overall vehicle [2]. The structure of a wheel hub primarily includes the rim and spokes: the rim is used to mount, support, and secure tires, while the spokes connect the rim to the wheel center, allowing it to withstand various loads [3]. The wheel hub must be designed to ensure stability and reliability under all driving conditions [4]. Bionics is an interdisciplinary field that designs artificial systems and products by studying the structures and functions of living organisms. In wheel hub design, bionics can provide inspiration to help designers create a hub structure that meets both functional requirements and aesthetic considerations [5,6].

The honeycombed structure in nature primarily experiences axial forces. Through continuous evolution, it maintains minimal internal stress and deformation under axial loads, ensuring the safety of the eggs within a honeycomb [7]. Honeycombed structures are commonly utilized for their lightweight and high-strength properties, making them suitable for conditions similar to those experienced by wheel hubs. The stress characteristics of honeycombed structures closely resemble those of wheel hubs under such conditions. Huang Rui [8] et al. divided bionic design into two thought processes: “top-down” and “bottom-up”. They extracted the morphological characteristics of biological prototypes and used these results to complete algorithmic shape generation within the logic of parametric design, verifying the feasibility of the scheme through finite element analysis. Chen Yiting [9] et al. used the lightweight, high-strength structure of an *Arachanthus siliceous* shell as a bionic template and employed the response surface method to optimize and design the optimal structure scheme for a bionic hub. Compared to pre-optimization, the overall structure was reduced by 8.4%, and the spoke weight decreased by 25.6%. Additionally, the prediction accuracy of the maximum equivalent stress of the structure using a BP neural network improved by 56.97% compared to the Workbench response surface method.

TongShuai Zhang [10] et al. utilized a central composite material design algorithm to obtain a hub parameter sensitivity map, identifying areas of stress concentration in the hub. Based on this, they optimized the hub shape through topological optimization, resulting in a 16% reduction in hub mass. Yang [11] et al. studied the simulation results of a hub under radial loads, discovering that stress concentrations easily occurred in areas such as the inner wheel edge, mid-section of the spokes, and rim seat, which adversely affected the hub’s fatigue life. Ding You [12] et al. combined structural discrete topological optimization with biomimicry to optimize drone wing structures, achieving a 12.34% reduction in weight, thus validating the feasibility of special wing designs for ultra-light applications. Zhang Zhifeng [13] et al. performed biomimetic optimization on a furrowing plough based on the unique morphological characteristics of a soil-dwelling mole cricket’s claws, resulting in a new structure that reduced soil stress by approximately 56% compared to the original design. Liu Yihua [14] et al. conducted topological optimization based on the bending stress results of a specific hub, finding that the primary areas for optimization the spokes and flange face, achieving a lightweight structure without actually altering the hub’s structure or stiffness.

Song Guiqiu [15] et al. used the ANSYS analysis platform for the radial fatigue simulation of a hub, simulating dynamic radial loads across 40 load steps to identify critical nodes for fatigue life analysis.

Wu Jiantao, Sun Li, and Liu Qingying [16] developed a growth algorithm using the Grasshopper plugin, applying it to hub design to create parametric biomimetic models with various configurations. CY Wang [17] studied the superior mechanical properties of a human tibia to design a novel crash box with enhanced energy absorption and impact resistance, providing a reference for crash box design and optimization. Wang [18] et al. employed linear cumulative damage theory to develop a fatigue life prediction model for a hub, estimating a lifespan of 448,200 revolutions, with a 5.6% error compared to the bending fatigue test result of 473,300 revolutions, confirming the model’s validity.

Li [19] et al. focused on automotive disc brakes, employing variable-density topological optimization to maximize stiffness and frequency, establishing a comprehensive objective function through compromise programming and gray relational analysis. Following improvements, the dynamic performance of the caliper and bracket was enhanced, with a total mass reduction of 11%. Lu [20] et al. used dynamic ANSYSn16.0 to simulate three typical conditions for control arms, extracting key load points as boundary conditions, and conducting topological optimization based on the results, leading to a secondary design that met the stiffness and strength requirements with a 16.5% weight reduction.

This paper will summarize the aforementioned literature and employ numerical simulation for a biomimetic design of honeycombed structures in hubs, ensuring that stresses at the spokes and their connection to the center meet the design specifications, while using gray relational analysis for multi-objective optimization and conducting static and bending fatigue life predictions.

## 2. Biomimetic Hub Materials and Analytical Methods

Finite element analysis (FEA) is a numerical computation method used in engineering and physics to simulate and analyze the behavior of complex objects under given conditions [21]. It divides continuous physical structures into small, finite elements to solve complex problems by defining boundary conditions and loading scenarios [22]. Numerical methods were employed to solve the equations for each element, allowing for an understanding of the behavior of the entire model [23]. The results were analyzed and visualized to assess the maximum stress and deformation of a hub [24]. FEA is particularly important in hub design, as it can predict performance during actual use, such as strength and fatigue life, ensuring the safety and reliability of a hub [25].

### 2.1. Establishment of a Finite Element Model

**Figure 1:**
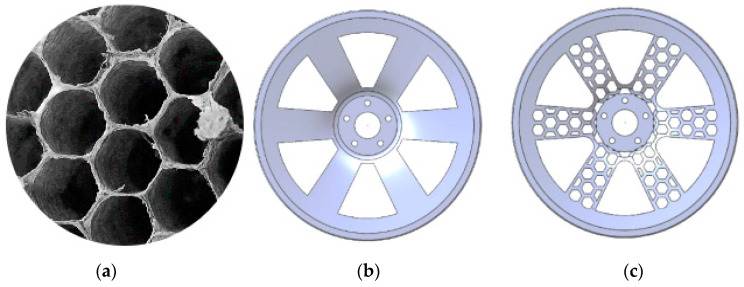
(**a**) Schematic diagram of a honeycomb structure; (**b**) Ordinary wheel hub; (**c**) Honeycomb-structured wheel hub.

In this paper, a new bionic lightweight wheel hub was designed based on a honeycombed structure, as illustrated in Figure 1a. This study referred to a 19-inch wheel hub to design and optimize the new bionic wheel hub, as depicted in the subsequent figure. Based on the stress distribution under bending conditions, the structure of the wheel hub was optimized, resulting in the bionic wheel hub shown in Figure 1b,c. For comparison, Figure 1b displays an ordinary wheel hub, while Figure 1c shows the honeycomb-structured wheel hub.

The wheel hub material used was A356 aluminum alloy, which is a ternary alloy with several advantages for hub applications. These include a low density, which helps reduce the weight of the hub, thereby improving overall vehicle fuel efficiency and handling. It also offers excellent mechanical properties: the alloy possesses high strength and rigidity, capable of withstanding various loads during vehicular operation. Additionally, A356 has outstanding casting performance, allowing for the production of complex hub shapes while minimizing casting defects and increasing production efficiency. These characteristics make A356 aluminum alloy an ideal choice for automotive wheel hubs.

The physical properties of the designed hub are shown in Table 1, and its strength met the design requirements of a wheel hub. The wheel hub material defaulted to a linear model. Because the tire will involve nonlinear problems in the finite element analysis, it was only necessary to analyze the wheel hub model, simplify the model to improve the mesh quality, and ensure the authenticity and accuracy of the simulation calculation [26].

The minimum fatigue cycle number was obtained through numerical simulation by applying a rotating bending moment to the fixed wheel hub. In Table 2, according to GB/T 5334-2021’s [27] “Passenger Car Wheel Performance Requirements and Test Methods”, the number of cycles required for the bending fatigue test was specified. The requirements and test methods for the passenger car wheel performance were designed to ensure the safety and comfort of the vehicle under various driving conditions [28], while wheel fatigue analysis evaluated the performance of the wheel during long-term use, providing a basis for the design and improvement in the wheel [29]. These requirements and methods were interrelated and together constituted a complete performance evaluation system for passenger car wheels, from design to use [30].

In finite element analysis (FEA), mesh independence analysis is a crucial step in ensuring the accuracy and reliability of simulation results. This analysis verified whether computational outcomes stabilized as the mesh was refined. If the results varied little with different mesh sizes, this indicated that the chosen mesh could provide reliable solutions. An excessively coarse mesh may have led to numerical errors and inaccurate predictions of stress concentrations, and mesh independence analysis could identify and eliminate these potential issues. By ensuring the accuracy of the results, this analysis aided in finding the optimal mesh division, balancing computational efficiency and precision while avoiding unnecessary resource waste. As shown in Table 3, when the number of elements reached 9.6 × 10^5^, the maximum stress value of the hub exhibited a stable trend; therefore, this mesh size was selected for subsequent numerical simulations.

### 2.2. Load Calculation under Bending Conditions

The load calculation of the wheel hub under bending conditions [31] included the calculation of the shaft end load and bolt preload. Formulas (1) and (2) were used for the calculation of the shaft end load.

(1) $$M=\left(\mu R+d\right){*F}_{V}*S$$

M: bending moment (Nm), μ (coefficient of friction between the tire and the road) = 0.7, R: static load radius of tires (m), d: deflection of the hub (m), F_v_: rated load (N), S: strengthening factor (optimization coefficient).

Shaft End Loads,

(2) F=M/L

L: (moment arm) = 0.7 m, F = 6311.7 N. F is the load at the end of the wheel hub shaft and the final calculation was 6311.7 N. In the calculation of the bolt preload under bending conditions [32], the wheel hub was fixed by connecting the bolt to the loading shaft during the experiment. Bolts with a torque up to 150 Nmde thread M14 × 1.5 were selected for the experiment, and the bolt preload was obtained using Formulas (3)–(7):

T_1_ is the ordinary thread moment,

(3) $$T_{1}=\frac{F_{Q}d_{2}}{2}\tan\left(\lambda +\rho_{v}\right)$$

F_Q_ is the bolt load of axial,

(4) $$F_{Q}=\frac{2T_{1}}{d_{2}\tan\left(\lambda +\rho_{v}\right)}$$

d_2_ is the diameter of the middle thread,

(5) $$d_{2}=d-0.6495\rho_{\nu }$$

λ is the angle of the thread,

(6) $$\lambda =\arctan\frac{\mathrm{np}}{{\pi d}_{2}}$$

$\rho_{\nu }$ is the thread angle of equivalent friction,

(7) $$\rho_{\upsilon }=\arctan f_{\nu }=\arctan\frac{f}{\cos\beta }$$

In the above formula, f (the friction coefficient of the nut-bearing surface) = 0.3, d is the large diameter of the bolt, d_2_ is the pitch diameter, n (number of threads) = 1, the pitch = 1.5, the common thread had a bevel angle of 30°, T_1_ is the bolt torque, and the calculation result was F_Q_ = 27,708 N.

The bending stress of a wheel hub in the process of driving is an instantaneous dynamic load [31], which is caused by road surface fluctuation during vehicular driving, the friction between the tire and road surface, and other dynamic load sources. In the process of static analysis, these dynamic loads need to be converted into static loads [32] in order to facilitate accurate calculation by using static equations. The theoretical basis of converting the dynamic load to the static load is to ensure the accuracy of the analytical model and the feasibility of calculation.

A wheel hub is constrained in two directions by the following: axial constraint and radial constraint. The hub typically has an axial constraint that prevents displacement during operation, keeping it in its proper position. The fit between the hub and the axle provides radial support, ensuring that the hub can withstand loads from the ground while maintaining the balance of the wheel’s rotation.

The essential difference between dynamic load and static load lies in the duration of load application and their characteristics. Dynamic loads are typically instantaneous and vary over time, whereas static loads are assumed to be constant throughout an analysis. When performing static analysis, it is necessary to convert dynamic loads into static loads, as static analysis assumes the load remains constant, allowing for the application of static equations in the calculations.

**Figure 2:**
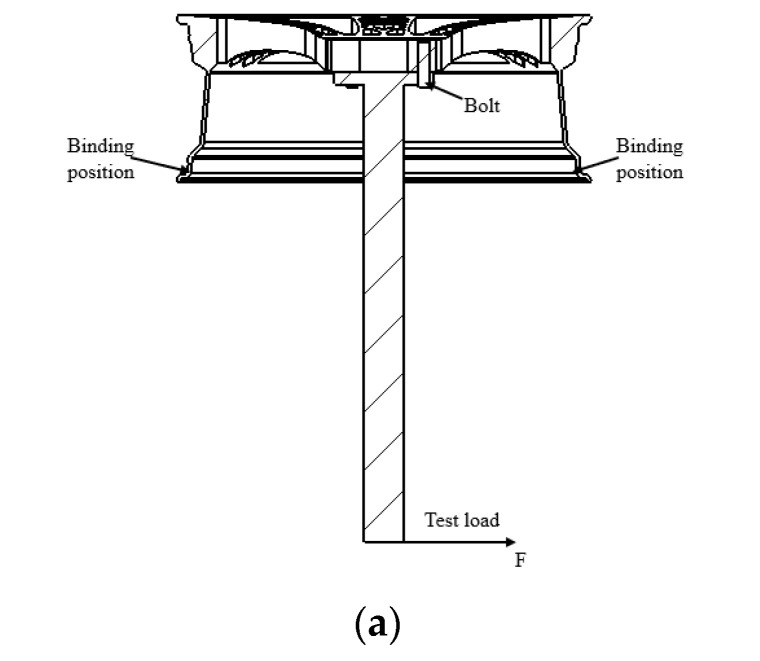

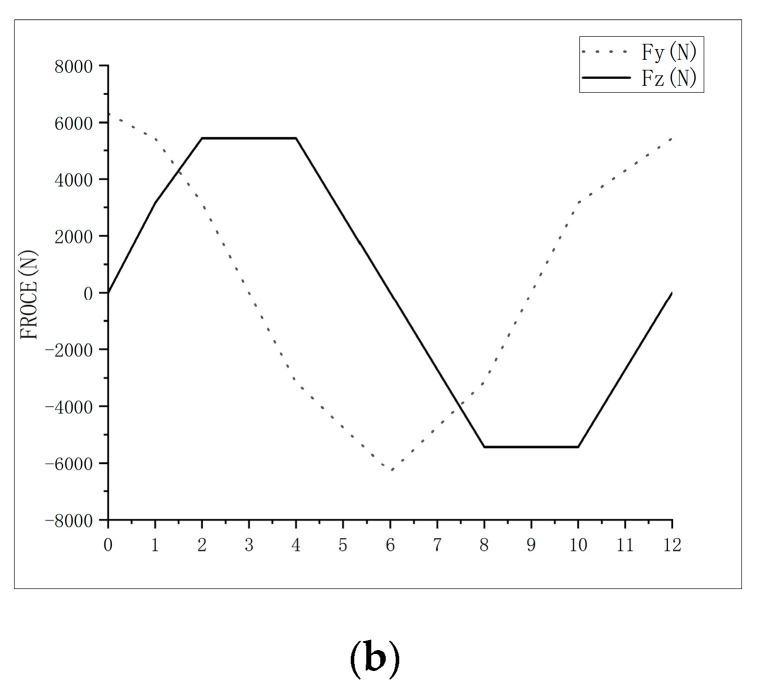
(**a**) Wheel hub stress diagram; (**b**) 12-load sequence values.

The end of a loading shaft is subjected to a bending moment, which is equivalent to the load at the end of the wheel hub shaft. The axial load is decomposed into component forces in the Z and Y directions. As shown in Figure 2b, this paper set 12 load sequences to simulate a complete loading process of the wheel hub, with an interval of 30° for the calculations.

### 2.3. Stress Comparison between the Bionic Wheel of the Honeycombed Structure and Ordinary Wheel Hub

According to the special symmetrical structure of the wheel hub, the following two working conditions were selected to compare the stress results. The first working condition was the force on the wheel hub being directed towards the center of the circle through the spoke of the wheel, while the second working condition involved the force being directed towards the center of the circle through two spokes [33]. Under condition 1 (where the force on the wheel hub was directed through one spoke towards the center of the circle), stress could be concentrated at the junction of the spoke and rim. In contrast, under condition 2 (where the force on the wheel hub was directed through two spokes towards the center of the circle), the location of the stress concentration could differ. These stress concentration areas are potential sites of fatigue failure; therefore, special attention should be given to the structural strength and fatigue life of these areas in the design of a wheel hub.

**Figure 3:**
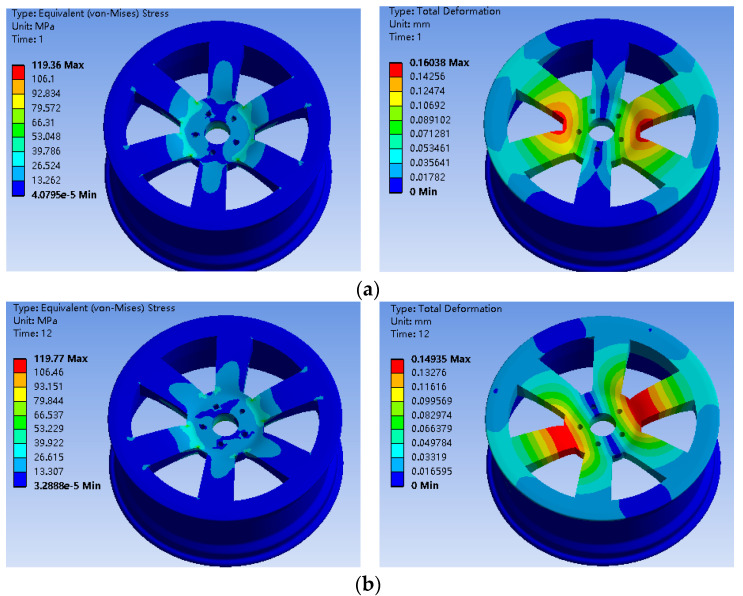

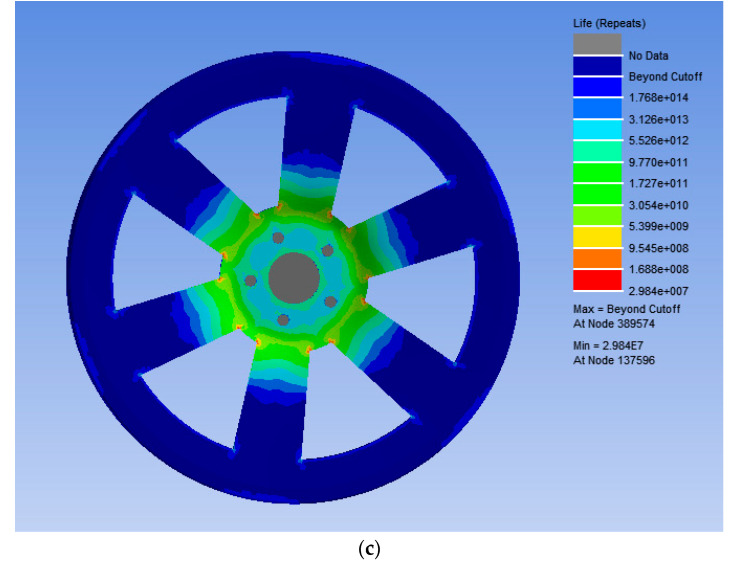
(**a**) Stress nephogram and displacement of the ordinary wheel hub under working conditions; (**b**) Stress nephogram and displacement of the ordinary wheel hub under working condition 2; (**c**) Fatigue life analysis results of the ordinary wheel hub.

**Figure 4:**
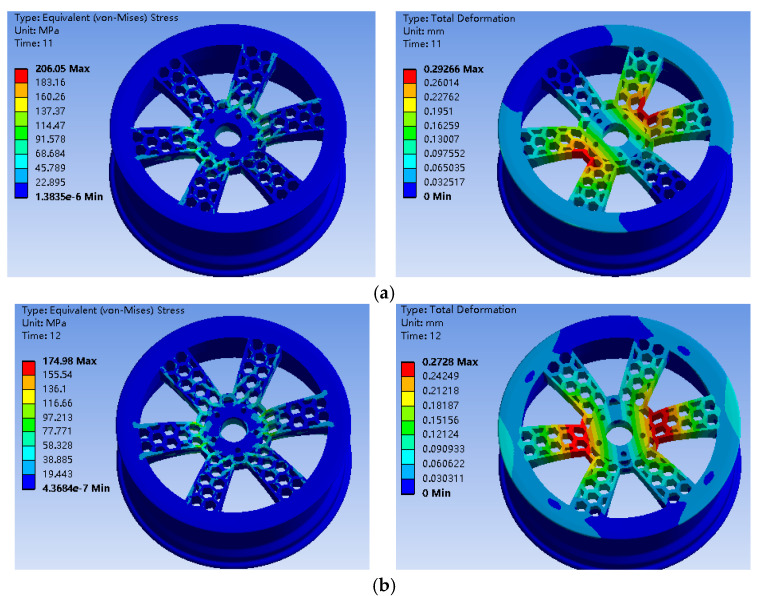

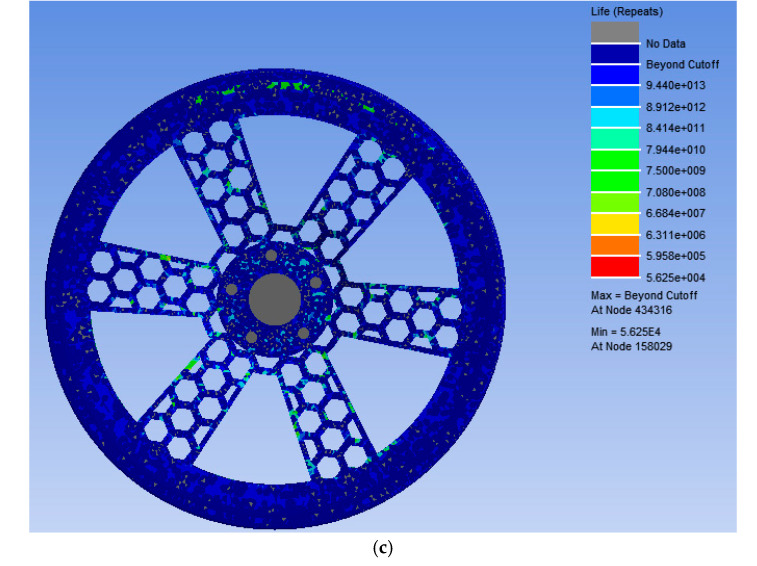
(**a**) Stress nephogram and displacement of the bionic wheel with a honeycombed structure under working condition 1; (**b**) Stress nephogram and displacement of the bionic wheel with a honeycombed structure under working condition 2; (**c**) Fatigue life analysis results of the bionic wheel with a honeycombed structure.

The finite element analysis results of the bionic wheel with a honeycombed structure and the ordinary wheel hub under two working conditions are shown in Figure 3 and Figure 4.

Under the two different working conditions, the maximum stress of the preliminarily designed bionic hub with a honeycombed structure was located near the center of the spoke. As shown in Table 4, the mass of the bionic wheel with a honeycombed structure was reduced by 4.29 kg compared to the ordinary wheel hub, with the spoke position reduced by about 30%. However, the maximum stress increased under the same working conditions. Although the wheel hub mass was reduced to some extent, the significant increase in stress resulted in a considerable decrease in the fatigue life of the wheel hub, which did not meet the optimization goals for the wheel hub [34].

## 3. Bionic Hub Structure Optimization Design of the Honeycombed Structure

The main function of the ring-reinforcing bar at the position of stress concentration in the wheel hub was to disperse stress and reduce the likelihood of fatigue cracks under repeated loading. Reinforcement can be considered a local strengthening measure that supports a wheel hub structure, reducing stress concentration caused by bending and thus extending its fatigue life.

**Figure 5:**
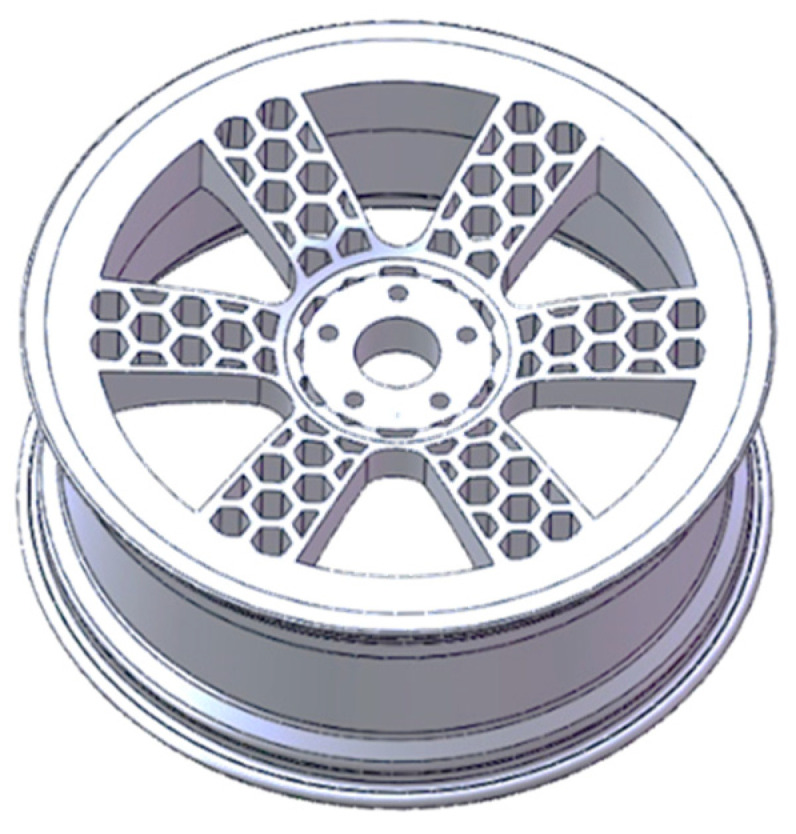
Bionic wheel hub with an optimized honeycombed structure.

To meet the requirements for the number of bending fatigue test cycles for the wheel hub, a ring reinforcement was added to the stress concentration area. The optimized model is shown in Figure 5. While ensuring strength and fatigue life, the lightweight design of the wheel hub structure was pursued as far as possible. The response surface optimization method [18] was employed to optimize the design of the middle ring stiffeners of the wheel hub.

### Response Surface Optimization Design of the Bionic Wheel Hub with a Honeycombed Structure

Response surface optimization (RSM) is a mathematical method for experimental design and parametric optimization. By establishing the response surface, a parametric space can be explored effectively to identify the parametric combination that allows a specific performance index to reach its optimal value. This approach is particularly useful in wheel hub design, as it enables designers to evaluate multiple design options within a limited number of experiments and determine which parameters are most critical to wheel hub performance [35,36].

**Figure 6:**
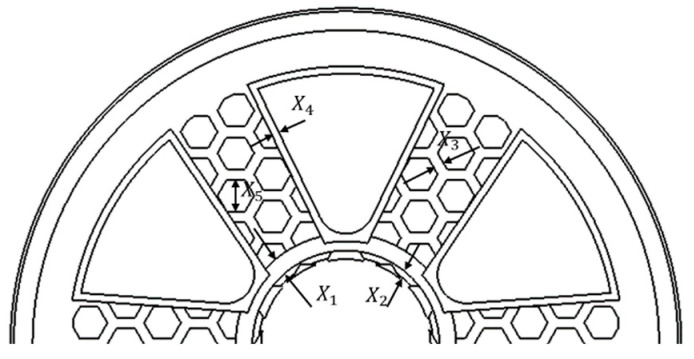
Distribution of the parametric structure corresponding to target optimization.

In the design, five parameters with a relatively low processing difficulty were initially selected. As shown in Figure 6, these parameters were the following: the thickness (*X_1_*) of the ring stiffener, the distance (*X_2_*) from the stiffener to the flange surface, the thickness (*X_3_*) of the spoke’s honeycombed wall, the thickness (*X_4_*) of the weight-reducing hole, and the diameter (*X_5_*) of the spoke’s honeycombed hole.

The design used the dx response surface optimization module to optimize the wheel hub structure. As shown in the figure above, five main size parameters were defined as optimization parameters, with the wheel hub stress and wheel hub mass as target parameters. As shown in Table 5, the variation ranges of the five parameters were defined.

#### Gray Correlation Degree Analysis of Five Parameters with Wheel Hub Stress and Quality

“Gray Correlation Analysis” [13] is a multi-factor statistical analysis method that identifies whether a certain degree of correlation exists between variables by analyzing the gray correlation degree of a data series [37,38]. In wheel hub design, this method helps analyze how different parameters of a wheel hub affect vehicular performance. In a gray system, the relative strength of wheel hub stress and mass, affected by five target parameters, can be obtained.

**Figure 7:**
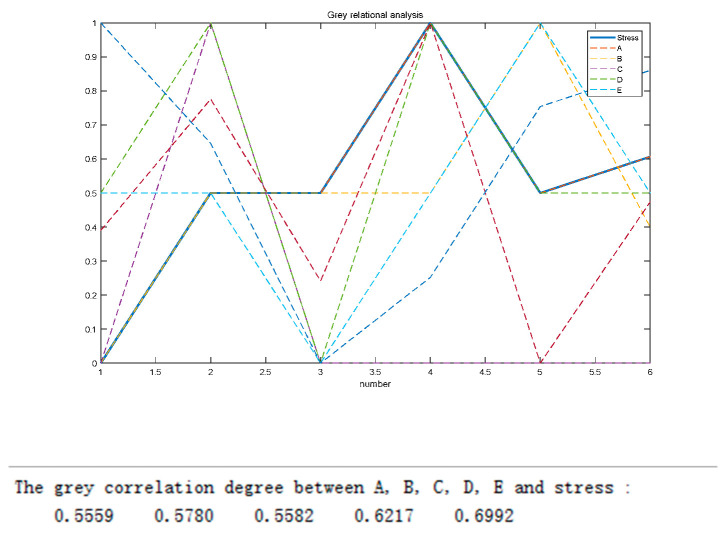
Gray correlation degree between target parameters and stress.

**Figure 8:**
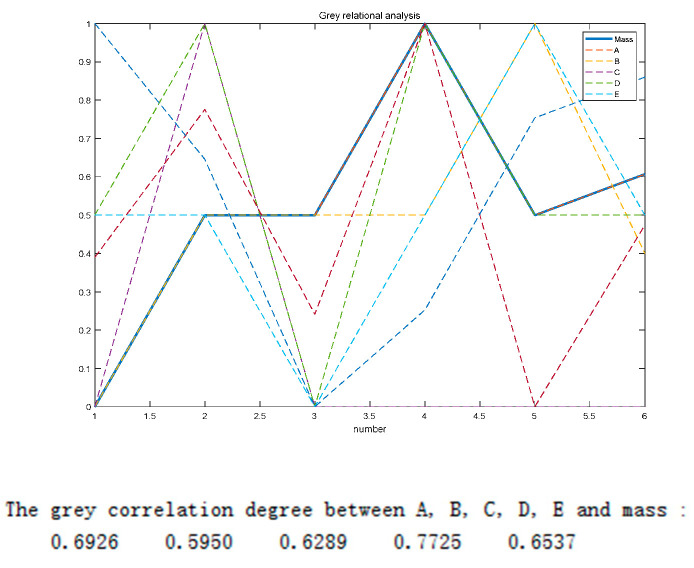
Gray correlation degree between target parameters and quality.

As shown in Figure 7, the correlation coefficients between the five parameter pairs and wheel hub stress were 0.5559, 0.5780, 0.5582, 0.6217, and 0.6692, respectively. The analytical results indicated that *X*_2_, *X*_4_, and *X*_5_ had a strong gray correlation with wheel hub stress. As shown in Figure 8, the correlation coefficients between the five parametric pairs and wheel hub quality were 0.6926, 0.5950, 0.6289, 0.7725, and 0.6537, respectively. The analytical results showed that *X*_1_, *X*_4_, and *X*_5_ had a strong gray correlation with wheel hub quality.

**Figure 9:**
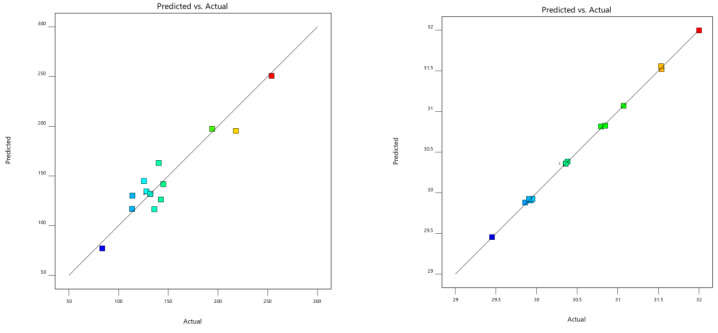
Fitting diagrams of the simulation results of the predicted value and actual value.

According to the linear regression model, Formula (8) is the linear regression equation of the wheel hub stress and five parameters, and Formula (9) is the linear regression equation of the wheel hub mass and five parameters. As shown in Figure 9, the predicted and actual values were distributed on both sides of the fitting line, which conformed to linear regression fitting.

(8) $$F=1665.58+256.95X_{2}-401.57X_{4}-84.11X_{5}+0.35X_{2}\cdot X_{4}+2.08X_{2}\cdot X_{5}-5.61X_{4}\cdot X_{5}-19.21X_{2}^{2}+40.11X_{4}^{2}+1.85X_{5}^{2}$$

(9) $$M=53.27+0.061X_{2}+0.55X_{4}-1.58X_{5}-0.03X_{2}\cdot X_{4}-0.002X_{2}\cdot X_{5}-\left(1.17E-16\right)X_{4}\cdot X_{5}+0.0006X_{2}^{2}-0.004X_{4}^{2}+0.023X_{5}^{2}$$

**Figure 10:**
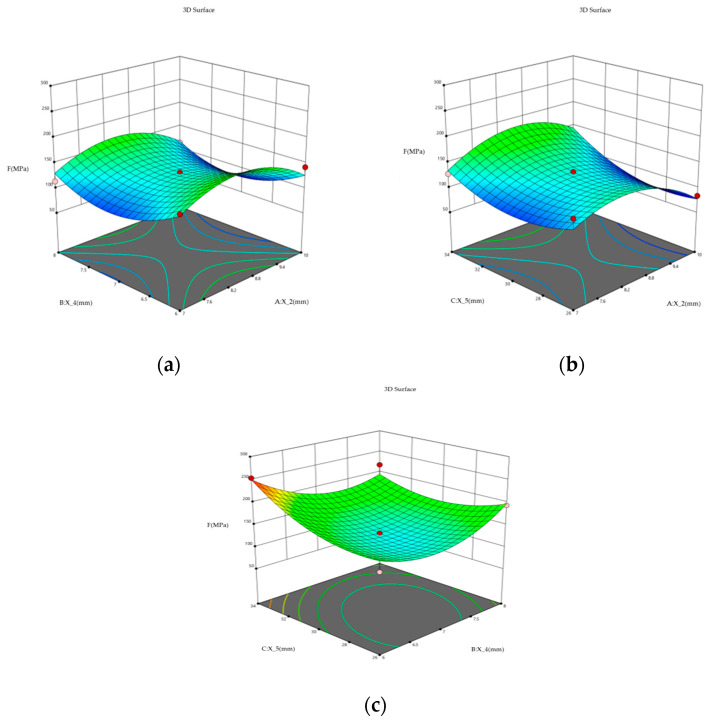
(**a**) Interactive effects of parameters *X*_2_ and *X*_4_ on stress values; (**b**) Interactive effects of parameters *X*_5_ and *X*_2_ on stress values; (**c**) Interactive effects of parameters *X*_5_ and *X*_4_ on stress values.

The first three parameters of the gray relational degree were determined by gray relational degree analysis as the distance from the stiffener to the flange surface *X*_2_, thickness of the weight-reducing hole *X*_4_, and diameter of the spoke’s honeycombed hole *X*_5_. As shown in Figure 10, the results show that when the two parameters changed at the same time [39], there was a point where the stress value was the smallest, and the value of the parameter corresponding to this point was designed, as *X*_2_ = 110.5 mm, *X*_4_ = 17.7 mm, *X*_5_ = 118.1 mm, so the bionic hub with an optimized honeycombed structure was designed and the simulation results were analyzed.

## 4. Results

**Figure 11:**
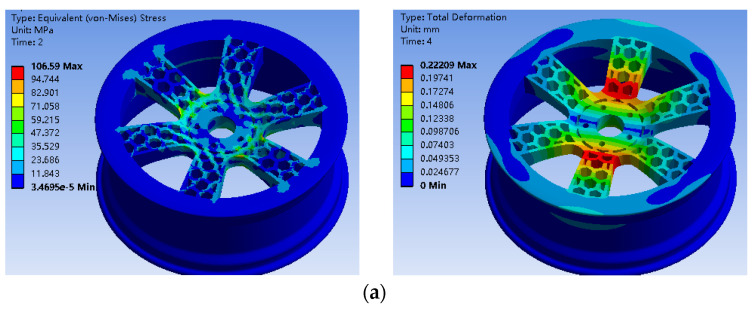

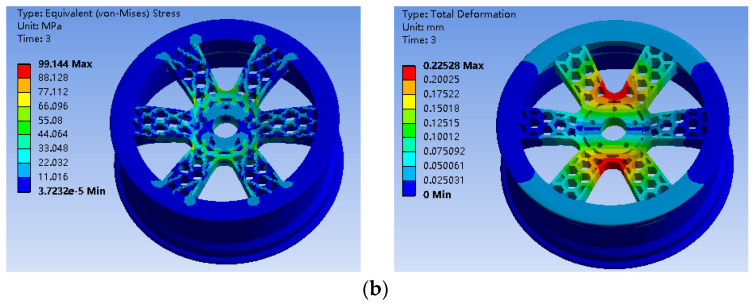
(**a**) Stress nephogram and displacement of the bionic wheel of the honeycombed structure after optimization under working conditions; (**b**) Stress nephogram and displacement of the bionic wheel of the optimized honeycombed structure under working condition 2.

**Figure 12:**
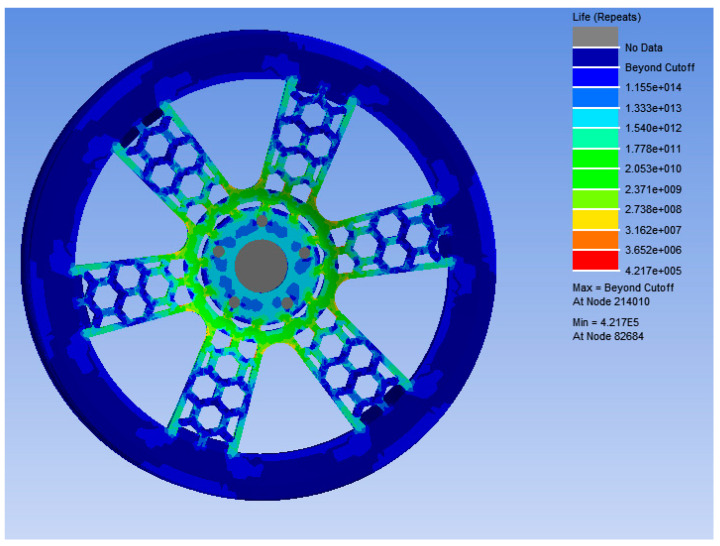
Fatigue life analysis results of the bionic wheel with an optimized honeycombed structure.

The stress nephogram and displacement of the bionic wheel of the optimized honeycombed structure under working condition 1 are shown in Figure 11a. The maximum stress occurred under working condition 1, with the maximum stress being 106.59 MPa and the maximum displacement being 0.22 mm. Under the second working condition, as shown in Figure 11b, the maximum stress of the bionic wheel of the optimized honeycombed structure was 99.144 MPa and the maximum displacement was 0.22 mm. As shown in Figure 12, it’s the fatigue life analysis results of the bionic wheel with an optimized honeycombed structure.

As shown in Table 6, the mass of the bionic hub with the optimized honeycombed structure obtained through finite element analysis was 31.17 kg. In comparison, the results from the response surface optimization analysis indicated that the mass was 29.89 kg, with an error of 4.2%. The maximum stress was 109.34 MPa, with an error of 2.5%, which met the error requirements of the optimization model.

The lightweight hub reduced the unstrung mass, improved the response of the suspension system, and enhanced vehicular stability and comfort. Additionally, the increased maximum stress value enabled the hub to withstand greater loads, thereby reducing the risk of damage and enhancing driving safety. By optimizing materials and design, this hub also decreased resource consumption, promoting sustainability. Overall, it had a significantly positive impact on performance, safety, and cost-effectiveness.

## 5. Conclusions

In this study, a wheel hub was optimized using two different optimization methods, aiming to improve its fatigue life and reduce weight. The optimization results showed that the maximum stress was reduced by 8.7%, and the wheel hub mass was decreased by 12.13% under the response surface optimization analysis method. Additionally, the bending fatigue life of the optimized honeycombed bionic wheel hub was predicted. The results indicated that under a stress of 109.34 MPa, the load cycles were at least 421,700, which met the minimum cycle requirements set by the national standard for wheel hub bending fatigue experiments. The main observations of this study were the following:

(1) The optimization results demonstrated that the maximum stress under the response surface optimization method was 109.34 MPa, which was 8.7% lower than the maximum stress of an ordinary wheel hub at 119.77 MPa. This reduction highlights the effectiveness of optimization in alleviating wheel hub stress.
(2) The mass of the ordinary wheel hub was 34.02 kg, whereas the mass of the optimized wheel hub was 29.89 kg, representing a reduction of 12.13%. The lightweight wheel hub not only improved fuel economy but also enhanced the vehicle’s handling performance.
(3) The bending fatigue life of the bionic wheel with the optimized honeycombed structure was predicted. Under a stress of 109.34 MPa, the load cycles would be at least 421,700 at the connection with the half-shaft. This exceeded the design requirement of a strengthening coefficient of 1.6 and met the minimum cycle number for bending fatigue tests of wheel hubs as specified in GB/T 5334-2021.

In summary, through the optimization of a honeycombed structure, the maximum stress of a wheel hub was significantly reduced, its weight was decreased, its the bending fatigue life was ensured to meet national standards. These optimization results not only demonstrate the effectiveness of the methods used but also provide strong support for performance improvement in a wheel hub.

## Figures and Tables

**Table 1:** Properties of A356 aluminum alloy.

Material	Density (kg/m^3^)	Young’s Modulus (Pa)	Poisson’s Ratio (MPa)	Strength Limit
A356 Aluminum alloy	2690	6.9 × 10^10^	0.33	290

**Table 2:** GB/T 5334-2021 “Passenger Car Wheel Performance Requirements and Test Methods” bending fatigue test cycle requirements.

Material	Strengthening Factor	Minimum Cycle Number	Friction Coefficient
Lightweight aluminum alloy	1.60 (optimization coefficient)	100,000	0.7

**Table 3:** Numbers of elements and maximum stress values of the hub.

Number of Grids	1.5 × 10^5^	1.9 × 10^5^	2.6 × 10^5^	3.7 × 10^5^	5.6 × 10^5^	9.6 × 10^5^	1.9 × 10^6^	4.3 × 10^6^
**Maximum Stress of the Hub (MPa)**	204.11	204.56	204.85	205.21	205.55	206.05	206.06	206.08

**Table 4:** Maximum stress and displacement values of the ordinary wheel hub and honeycomb-structured wheel hub under two working conditions.

Working Condition	Mass (kg)	Maximum Stress (MPa)	Displacement (mm)
Ordinary wheel hub 1	34.02	119.36	0.16
Honeycomb-structured wheel hub 1	29.73	206.09	0.29
Ordinary wheel hub 2	34.02	119.77	0.15
Honeycomb-structured wheel hub 2	29.73	174.94	0.27

**Table 5:** Variation ranges of target parameters.

Target Parameter	Variation Range (mm)
$X_{1}$	14–17
$X_{2}$	7–10
$X_{3}$	8–12
$X_{4}$	6–8
$X_{5}$	26–34

**Table 6:** Comparison of analytical results of the finite element analysis optimization and response surface optimization.

	Finite Element Analysis	Response Surface Optimization Design	Error
Mass (kg)	31.17	29.89	4.2%
Stress (MPa)	106.59	109.34	2.5%

## Data Availability

The data presented in this study are available on request from the corresponding author.
